# Type 1 Diabetes and Multiple Sclerosis Share General Autoimmunity Genetic Variation

**DOI:** 10.3390/genes17050531

**Published:** 2026-04-30

**Authors:** Maristella Steri, Alessandro Testori, Valeria Orrù, Magdalena Zoledziewska

**Affiliations:** 1 Institute of Genetic and Biomedical Research (IRGB), Italian National Research Council (CNR), 09042 Monserrato, Italy; annamaristella.steri@cnr.it (M.S.); valeria.orru@cnr.it (V.O.); 2 Institut für Genetische Epidemiologie, Universitäts Medizin Göttingen, 37073 Göttingen, Germany; alessandro.testori@irgb.cnr.it

**Keywords:** type 1 diabetes, multiple sclerosis, colocalization, autoimmunity, expression, expression quantitative loci, eQTL, pQTL

## Abstract

**Background/Objectives**: Type 1 diabetes (T1D) and multiple sclerosis (MS) are autoimmune, multifactorial, organ-specific disorders mediated by immune cells. Their co-occurrence has been partially attributed to shared genetics and environmental factors. We aimed to dissect the shared genetic architecture between T1D and MS using large-scale genome-wide association studies (GWASs) and colocalization analyses. **Methods**: We applied a Bayesian colocalization framework to two large-scale GWAS data sets: a T1D study comprising 18,942 cases and 501,638 controls, and an MS GWAS including 14,802 cases and 26,703 controls. **Results**: We identified 26 shared colocalizing association signals between T1D and MS. Among them, seven loci (*EOMES*, *RGS14*, *DLL1*, *ZNF438/ZEB1*, *SESN3*, *WARS1/SLC25A47*, and *IRF8*) were novel for T1D and two (*UBAC2* and *LAT*) for MS. Several signals showed supportive evidence in additional datasets and demonstrated functional annotation characteristics consistent with disease involvement. **Conclusions**: Colocalization can be a powerful discovery tool for disorders with co-divided genetic architecture, as prioritizing shared rather than individual causal variants may enhance the detection of novel loci. Our findings indicate that T1D and MS predominantly share general autoimmune susceptibility signals (17/26), rather than disease-specific (private), often with opposite direction of effect (9/26), underscoring their immunological heterogeneity.

## 1. Introduction

Type 1 diabetes (T1D) and multiple sclerosis (MS) are autoimmune, organ-specific, immune cell-mediated disorders.

All autoimmune disorders share a genetic component. Based on this expectation, we applied statistical colocalization to the two largest genome-wide association studies (GWASs) available until now, in order to detect novel associations, which we then replicated using additional publicly available datasets.

A similar approach was used during the development of the Immunochip, an array targeting 196,000 single-nucleotide polymorphisms (SNPs) derived from genome-wide association studies of 11 autoimmune disorders. These results were then used to establish the shared genetic component between autoimmune diseases and to expand the number of candidate shared associations [1,2]. The genetic overlap between T1D and MS is only partially known, although the Immunochip-based study has highlighted that shared allele risk of autoimmune disorders including T1D and MS also influences gene expression in different cell types [1].

As it was observed for the majority of autoimmune disorders, T1D and MS also co-occur, with a three-fold higher incidence of MS reported in T1D patients [3]. Furthermore, it has been estimated that 8.6% of patients with MS have at least one comorbid autoimmune disorder, with 1.7% having also T1D [4]. T1D and MS are both complex, autoimmune disorders that involve loss of immune tolerance and share a fraction of genetic components, although their organ specificity and clinical manifestation differ (Table 1). Such differences in co-occurring disorders may be the cause of a large portion of antagonistic pleiotropy. To study the mechanisms of antagonistic pleiotropy of autoimmune disorders, we chose these two diseases, which exhibit various genetically determined clinical differences. Furthermore, studying the antagonistic pleiotropy and genetic sharing may help in drug repositioning and explain their autoimmune side effects.

Colocalization analyses investigate whether two traits or diseases share a common causal variant at a given locus, rather than simply quantifying genome-wide shared heritability, as is done by other methods such as genetic correlation and cross-trait LD score regression [5]. This locus-specific analysis provides high resolution for identifying shared genetic signals and offers great insight into underlying biological mechanisms, while reducing the risk of confounding due to linkage disequilibrium.

It has also been shown that various genetic risk loci are shared across autoimmune disorders [1,6,7,8,9,10]. Co-segregation of various immune-mediated disorders within families affected by autoimmunity further suggests a general susceptibility to autoimmunity [11,12]. However, families also share the same environment, geographic location, diets and environmental exposures. Thus, the shared genetics of different autoimmune disorders mirrors their shared etiology. Joint likelihood mapping across six autoimmune diseases identified both shared and distinct association signals, and despite the widespread sharing of pathogenic mechanisms, no global mechanism has been characterized for autoimmune polygenic disorders [1].

T1D and MS are both complex, cell-mediated, autoimmune disorders that involve loss of immune tolerance and share a fraction of genetic components, although their organ specificity and clinical manifestations differ. There have been no clear reports detailing their genetic overlaps.

Both disorders show heterogeneity [13], partially attributed to human leukocyte antigen (HLA) class II genotypes [14]. Several genes outside the HLA region act as susceptibility loci in both T1D and MS, but have modest effect sizes.

HLA class II haplotypes involved in antigen presentation are responsible for about 50% of heritability in T1D [15]. A total of 136 non-HLA loci have been described for T1D [16], while for MS, 201 variants outside and 32 within the extended MHC region were discovered [17].

We previously reported shared genetic regulation between those two disorders at key autoimmunity loci. For instance, a variation in *TYK2* was found to have a protective effect in MS and T1D, while variants in *CLEC16A* predispose to both disorders. In contrast, variation in *PRF1* showed the opposite direction of effects [18,19,20].

Here, we describe two distinct etiopathogenic mechanisms underlying the two diseases, which share several general autoimmune susceptibility loci, some of which exert opposite effects on the risk of the two disorders. Thus, the two diseases etiologically and mechanistically share only a part of the general autoimmunity similarities, and often with contrasting effects. Nevertheless, identifying a shared origin of comorbidities contributes to the understanding of disease mechanisms, treatment development, and patient stratification for therapeutic intervention.

## 2. Materials and Methods

We performed a colocalization study between two publicly available GWAS datasets of T1D and MS to reveal shared genetic associations, selecting loci that showed suggestive evidence of association (down to *p* value < 10^−5^) in both diseases. Next, we searched for the replication of the novel variants in the independent publicly available datasets. Additionally, we integrated publicly available expression quantitative trait loci (eQTL) and protein quantitative trait loci (pQTL) data to perform pathway analysis, with the aim of providing an etiological/functional interpretation to the colocalizing signals.

For colocalization analyses, two large data-sets were used: (i) the T1D GWAS based on 18,942 cases and 501,638 controls from the Chiou J et al. 2021 Nature study (NHGRI-EBI GWAS catalog, accession number GCST90014023) [16]; (ii) the MS GWAS discovery study of the International Multiple Sclerosis Genetics Consortium 2019 Science publication of 14,802 cases and 26,703 controls (obtained upon request from the IMSGC website, http://imsgc.net) (final summary statistics were not available) [17]. Both studies involve mainly European samples, with a smaller admixture of Asian and African populations.

Colocalization was conducted using the *coloc.abf* function from the *coloc* package (version 2.1) in R (version 4.3.1), using the default prior probabilities (*p*1 = 1 × 10^−4^, *p*2 = 1 × 10^−4^, *p*12 = 1 × 10^−5^) [21]. Considering the different genome builds used in the two studies (MS in GRCh37 and T1D in GRCh38), we aligned the T1D data to the former. All variants located within a region extending from 100 kb upstream of the lead variant with the lowest genomic coordinate in the GRCh37 build to 100 kb downstream of the second lead variant were included. We considered PP.H4 ≥ 0.8 as strong evidence of colocalization, and values between 0.7 and 0.8 as suggestive.

To establish the direction of effects, linkage disequilibrium (LD), quantified as the square correlation coefficient (r^2^), was first calculated to assess the correlation between the lead variants. When r^2^ ≥ 0.20, phasing was subsequently performed to align alleles across datasets and identify coupled alleles. LD and phasing were calculated using PLINK v1.9 based on European samples from the 1000 Genomes Project. Variant positions were reported in the GRCh37 genome build.

Additional information on the variants’ functional characteristics was recovered from e!Ensembl (http://ensembl.org) and UCSC Genome Browser (http://genome.ucsc.edu/, accessed on 20 April 2026). In particular, e!Ensembl was used to establish ancestral allele and variant frequencies, while the UCSC Genome Browser was interrogated to define the functional effects of colocalizing variants.

We searched for the replication of the novel variants in additional publicly available datasets: for T1D, we retrieved summary statistics from the Type 1 Diabetes Knowledge Portal (http://t1d.hugeamap.org, accessed on 20 April 2026), while for MS, we used summary statistics provided by De Jager P and Kiryluk K [22] (personal communication). In particular, we considered an association to be replicated if it had a *p* value < 0.05. The T1D Knowledge Portal was also used to search for associations with other diseases or quantitative traits.

The Open Targets Platform (https://platform.opentargets.org, accessed on 20 April 2026) was interrogated to search for molecular QTLs (eQTLs, pQTLs). This tool integrates publicly available datasets, including data generated by the Open Targets consortium. It integrates relevant annotation information about targets, diseases or quantitative phenotypes, genetic variants, GWASs and drugs as well as their most relevant relationship.

The UKB PPP EUR proteomic database was queried from the Open Targets Platform tool. The UKB-PPP is a collaboration between the UK Biobank (UKB) and thirteen biopharmaceutical companies, characterizing the plasma proteomic profiles of 54,219 UK participants [23]. Detailed information on the criteria used to define a SNP as a pQTL is described in Sun et al.’s publication [23].

The Interval portal (https://IntervalRNA.org.uk) was used to identify potential eQTLs, sQTLs and corresponding pQTLs. This platform integrates multi-omics data from the INTERVAL study and external cohort, enabling the investigation of gene-regulatory mechanisms and including colocalization analyses with proteomic and metabolomic traits to assess shared genetic signal [24].

Pathway analysis was conducted by ShinyGO 0.85.1: a graphical gene-set enrichment tool for animals and plants (https://bioinformatics.sdstate.edu/go/, accessed on 20 April 2026) [25]. KEGG diagrams were drawn with KEGG and Pathview [26,27].

Enrichment analysis gives a graphical gene-set enriched GO terms and other pathways for over 14,000 species based on annotation from the Ensembl and STRING-db databases.

Network analysis was drawn by the Shiny GO tool which produces an interactive plot showing the relationship between enriched pathways. Two pathways are connected if they share more than 20% of genes. Darker nodes are significantly enriched gene sets, and bigger nodes represent larger gene sets. A hierarchical clustering tree summarizes the correlation among significant pathways listed in the Enrichment table. Pathways with many shared genes are clustered together.

Regional association plots displaying association statistics and local linkage disequilibrium in each genomic region were drawn using the standalone version of LocusZoom v1.4 [28]. Linkage disequilibrium was calculated using the European samples, including the CEU, GBR, FIN, TSI, and IBS subpopulations, from the 1000 Genomes Project phase 3 (https://www.internationalgenome.org/data/, accessed on 20 April 2026).

## 3. Results

### 3.1. Colocalization of T1D and MS Genetic Association Data and Functional Annotation of the Associated Variants

We used the two largest available GWASs for MS and T1D [16,17]. We first identified suggestive association signals (*p* value < 1 × 10^−5^) in each disease and focused on overlapping loci showing evidence of association in both datasets. For these shared loci, we applied a colocalization approach to assess whether the same underlying genetic variants contributed to risk in both diseases, with the aim of identifying novel shared genetic risk factors. We then evaluated the robustness of these findings by replicating the analyses at a nominal threshold (*p* < 0.5) using additional publicly available GWAS summary statistics.

We found 26 colocalizing association signals between T1D and MS, seven of which were novel associations for T1D (*EOMES*, *RGS14/PRLD1*, *DLL1/FAM120B*, *ZNF438/ZEB1*, *SESN3/MTMR2*, *WARS1/SLC25A47* and *IRF8*) and two for MS (*UBAC2* and *LAT*) (Table 1). These signals have functional annotation characteristics linked to those diseases.

Interestingly, 17 signals showed the same direction of effect in both diseases, whereas 9 signals had opposite effects, indicating that some loci may act through common biological pathways, whereas others exert antagonistic effects across diseases.

Moreover, seven signals colocalized and thus shared the same causal variants, but their lead variants were only weakly correlated (r^2^ < 0.2), indicating the presence of genetic allelic heterogeneity at the locus. This means that multiple variants at the same locus contribute to the association signals, complicating fine-mapping and causal variant prioritization.

Most of the tested variants were not distinctive for MS and T1D, because they were also associated with other autoimmune disorders, indicating a tendency toward general autoimmunity rather than disease-specific effects, according to the public databases Open Targets Platform and UCSC-Genome Browser.

The novel findings—particularly those supported by high linkage disequilibrium between lead variants (r^2^ > 0.5) and functional evidence indicating a regulatory role of the variant—are described below.

#### 3.1.1. Novel T1D Associations Colocalizing with Known MS Signals

**Figure 1:**
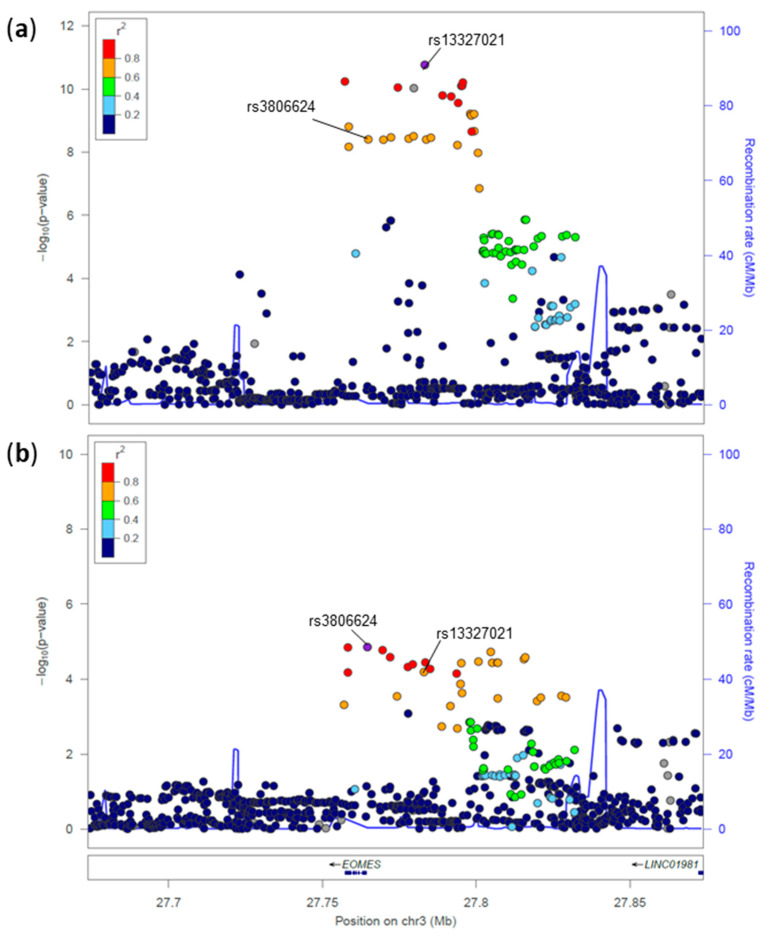
Regional association plot for the *EOMES* locus in MS (**a**) and T1D (**b**). Each point represents a genetic variant plotted according to its genomic position (*x*-axis) and −log10(*p*-value) of association (*y*-axis). The lead variant is shown as a purple diamond, while surrounding variants are colored based on their linkage disequilibrium (r^2^) with the lead variant, calculated using European samples from the 1000 Genomes Project. Recombination rates (cM/Mb) are shown as a blue line on the secondary *y*-axis. Gene annotations and transcriptional orientation are displayed below the association signals. Variant positions are based on the GRCh37 genome build.

**Figure 2:**
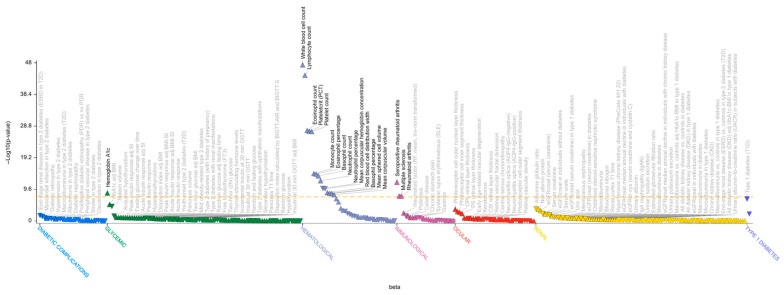
The Phenome-wide association (PheWAS) plot of the rs3806624*A variant generated with T1D Knowledge Portal. The PheWAS plot shows the significant (*p* value ≤ 0.05) associations of rs3806624*A for all available traits in the T1DKP, generated by bottom-line integrative analysis across all datasets in the Portal. The triangle data points indicate direction of effect and group-related traits on (*x*-axis) and −log10(*p*-value) of association is presented on the *y*-axis.

The novel T1D-associated variant rs3806624*A (Table 2), located in the intergenic region of the eomesodermin (*EOMES*) gene, which encodes the T-box transcription factor, represents the most interesting finding. The rs3806624*A is associated with increased risk of T1D (*p* = 1.4 × 10^−5^, OR = 1.06, 95% CI: 1.03–1.09) [16] and replicates nominally in two additional T1D studies [29,30]. At the same locus, the previously reported variant rs13327021*C had an opposite effect in MS compared to the T1D novel signal led by rs3806624*A (Table 1; Figure 1 and Figure 2). These two lead variants are in LD in the European population (r^2^ = 0.685), whereas they are independent in the African population (r^2^ = 0.051). Together, these findings suggest heterogeneity of genetic effects at the *EOMES* locus in T1D and MS.

**Figure 3:**
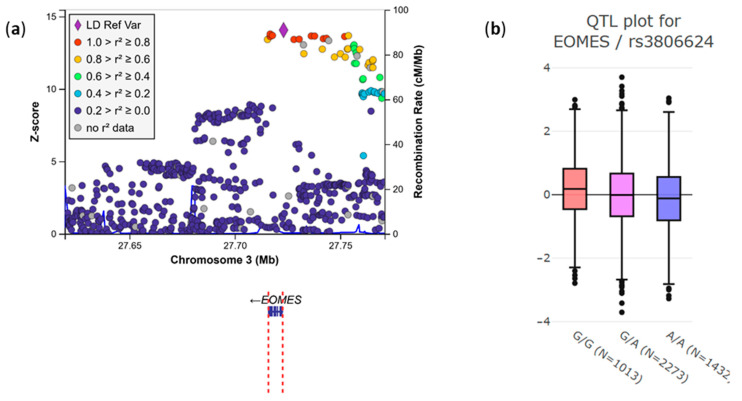
rs3806624 eQTL in the *EOMES* gene according to the INTERVAL study. (**a**) Each point represents a genetic variant plotted according to its genomic position (*x*-axis) and association Z-score (*y*-axis), where positive values indicate effect estimates in the direction of the tested allele and larger absolute values indicate stronger statistical evidence. The lead variant is shown as a purple diamond, while surrounding variants are colored based on their linkage disequilibrium (r^2^) with the lead variant, calculated using European samples from the 1000 Genomes Project. Recombination rates (cM/Mb) are shown as a blue line on the secondary *y*-axis. Gene annotations and transcriptional orientation are displayed below the association signals. Variant positions are based on the GRCh38 genome build. The SNPs rs3806624 (violet triangle) and rs2887944 are in high LD and closely located in the plot. (**b**) Boxplot of the EOMES expression stratified by rs3806624 genotypes. Each box spans from quartile 1 to quartile 3; a line inside the box marks the median, whereas “whiskers” extend to the minimum and maximum.

Interestingly, the rs3806624*A variant is in strong LD (r^2^ = 0.9) with the functional variant rs2887944*G located in the 3’UTR of the *EOMES* gene and falls into the promoter of the gene. Variant rs2887944*G is an eQTL associated with decreased *EOMES* expression (*p* = 2.28 × 10^−42^, Z-score = 13.78), according to the INTERVAL browser (Figure 3) [24].

Notably, rs2887944*G shows differences in allele frequency across populations, being more frequent in Europeans (MAF = 0.55) than in Africans (MAF = 0.21) or East Asia (MAF = 0.16).

Another novel association with T1D is localized in the *RGS14/PRLD1* gene region (Supplementary Figure S1). In detail, variant rs67111717*A, previously associated with MS, is in partial LD (r^2^ = 0.405) with rs4073745*A, a novel variant showing suggestive association with T1D (*p* = 2.30 × 10^−7^, OR = 1.09, 95% CI: 1.05–1.12) [16], and suggestively replicated in a partially independent large study of individuals of the same European ancestry [30]. Both variants have the same direction of effects. The first variant is located in the regulator of G protein signaling 14 (*RGS14*) gene, whereas the second maps in the PRELI domain-containing protein 1, mitochondrial (*PRLD1*) gene.

In the *DLL1/FAM120B* gene region, the previously reported MS-associated signal, led by rs2273215, colocalized with a novel suggestive signal for T1D, led by rs1075163 (Supplementary Figure S2). Notably, these variants have the opposite direction of effects in the two diseases. The rs1075163*G variant, relatively common in Europeans (MAF = 0.11) was suggestively associated with T1D (*p* = 8.96 × 10^−6^, OR = 0.91, 95% CI: 0.87–0.95) [16]. This variant suggests replicability in an independent study conducted in a population of the same ancestry [30]. The variant is located in the *FAM120B* gene with unknown function.

Still, in the *ZNF438/ZEB1* gene region, the previously reported MS association, led by rs1087056, colocalized with a novel, independent signal for T1D, driven by variant rs2066250*C (*p* = 2.37 × 10^−6^, OR = 1.08, 95% CI: 1.05–1.11) (Supplementary Figure S3). Both signals map to the long non-coding RNA LINC02664, located within the *ZNF438* gene region. The T1D association is suggestively replicated in a partially independent study of individuals of European descent [30]. Interestingly, the *ZEB1* gene, localized in the associated region, encodes a transcriptional repressor known to inhibit interleukin-2 (IL-2) gene expression.

Another novel association with T1D is located in the *SESN3/MTMR2* gene region, in which variant rs11021232*C, suggestively associated with T1D (*p* = 1.1 × 10^−5^, OR = 1.08, 95%CI: 1.05–1.12) [16], is also suggestively replicated in the FinGen Data Freeze 2 study (https://www.fingen.fi/en, accessed on 20 April 2026; FinnGen 2018 GWAS: European ancestry). The top MS colocalizing variant, rs4409785*C, is in high LD with the leading T1D-associated variant (r^2^ = 0.95, Supplementary Figure S4) and shows the same direction of effects. The T1D association was suggestively replicated for T1D in an independent study of the same ancestry [31]. Functionally, rs4409785 acts as an eQTL primarily in CD4-positive, alpha-beta T cells, in which the risk allele reduces expression of sestrin 3 (*SESN3*) (*p* = 8.73 × 10^−37^, beta = −0.601, se = 0.0454; Open Targets Platform: https://platform.opentargets.org/variant/11_95578258_T_C, accessed on 20 April 2026). Conceivably, rs11021232 also exerts a similar molecular effect, being reported as an eQTL that decreases the expression of the *SESN3* gene in naïve regulatory T cells, CD4-positive T lymphocytes, alpha-beta T cells, and LNCRNA-IUR in blood (Open Targets Platform: https://platform.opentargets.org/variant/11_95587644_T_C). *SESN3* encodes sestrin 3, a protein that reduces the levels of intracellular reactive oxygen species. Through its antioxidant activity, sestrin 3 contributes to lowering the levels of ROS detoxification [32].

**Figure 4:**
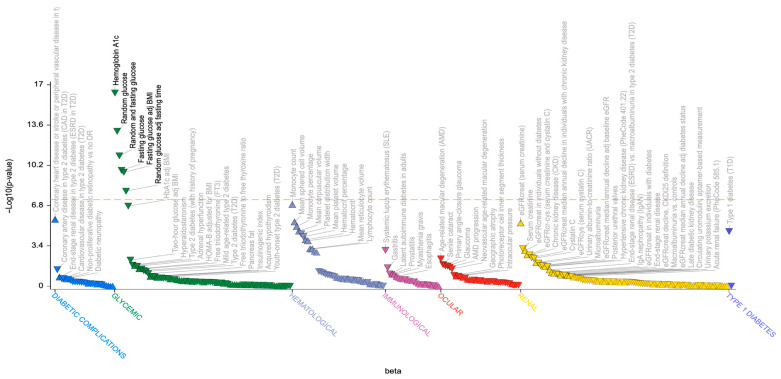
The Phenome-wide association (PheWAS) plot of the rs8009765*A variant. The PheWAS plot shows the significant (*p*-value ≤ 0.05) associations of rs8009765*A for all available traits in the T1D Knowledge Portal (T1DKP), generated by bottom-line integrative analysis across all datasets in the Portal. The triangle data points indicate the direction of effect. Related traits are grouped on the *x*-axis, whereas the statistical significance of association (expressed as −log10(*p*-value)) is indicated on the *y*-axis.

In the *WARS1/SLC25A47* gene region, the rs8009765*A variant, associated with MS in the exploratory study but only minimally supported in the large meta-analysis [17], colocalized with a signal whose lead, synonymous variant rs8015259*G, shows a suggestive association with T1D (*p* = 7.1 × 10^−5^, OR = 1.07, 95% CI: 1.03–1.11, Supplementary Figure S5) [16]. This association was suggestively replicated in the FinnGen GWAS conducted in individuals of European ancestry [33]. According to the T1D Knowledge Portal (t1d.hugeamap.org), rs8009765 is associated with several glycemic traits related to diabetes, including hemoglobin A1c and fasting glucose traits (Figure 4).

**Figure 5:**
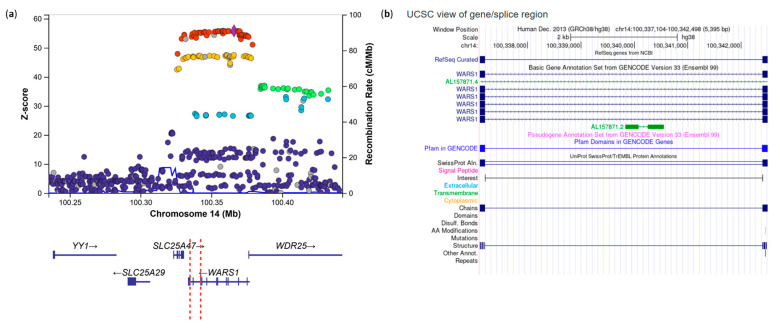
rs724391 QTL. (**a**) Regional association plot of the expression QTL in the region of the *SLC25A47/WARS1* gene region. Each point represents a genetic variant plotted according to its genomic position (*x*-axis) and association Z-score (*y*-axis). The lead variant is shown as a purple diamond, while surrounding variants are colored based on their linkage disequilibrium (LD; r^2^) with the lead variant, calculated using European samples from the 1000 Genomes Project. Recombination rates (cM/Mb) are shown as a blue line on the secondary *y*-axis. Gene annotations and transcriptional orientation are displayed below the association signals. Variant positions are based on the GRCh38 genome build. (**b**) The UCSC Genome Browser view of the spliced region of exon 10 of the *WARS1* gene, according to the INTERVAL browser.

The rs8015259 variant is in strong LD with rs724391*C (r^2^ = 0.95), a variant associated with decreased excision of exon 10 and increased plasma tryptophanyl-tRNA synthetase (*WARS1*) protein levels. Exon 10 of *WARS1* encodes the portion of the tRNA synthetase domain. Notably, WARS1 is regulated by IFN-γ (Figure 5).

rs8015259 is an eQTL for both the *WARS1* and *SLC25A29* genes in several immune cell subpopulations. Although its association with MS is weak, the variant is linked to decreased expression of the *WARS1* in the cerebellum and C1 segment of the cervical spinal cord (Open Targets Platform).

**Figure 6:**
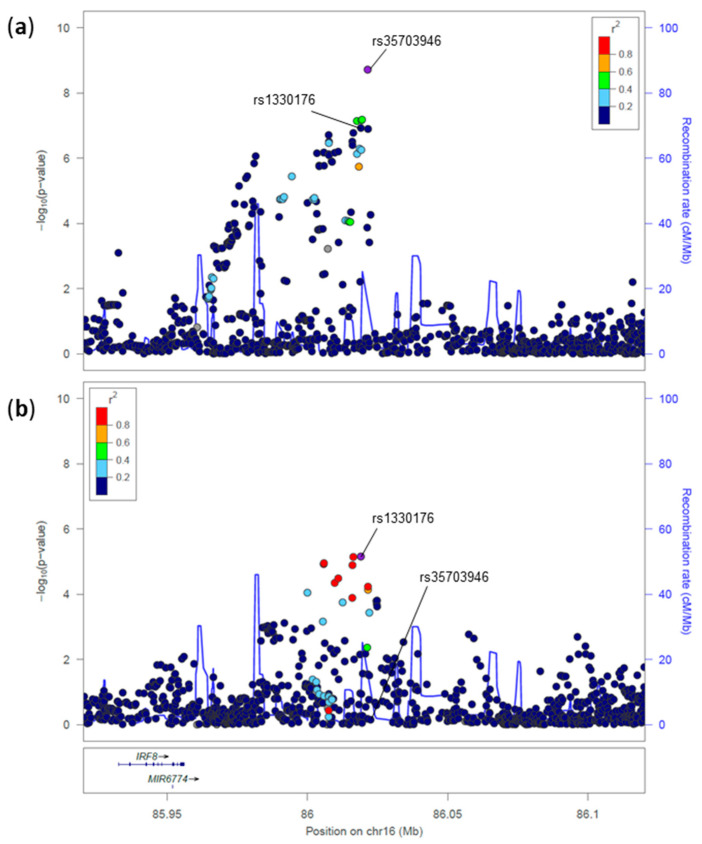
Regional association plot for the *IRF8* locus in MS (**a**) and T1D (**b**). Plot details, including variant coloring by linkage disequilibrium (r^2^), recombination rates, and gene annotations, are described in Figure 1.

**Figure 7:**
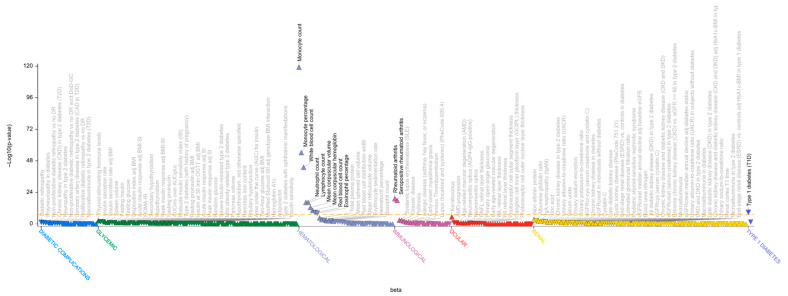
The Phenome-wide association (PheWAS) plot of the rs13330176*A variant generated with T1D Knowledge Portal. The PheWAS plot shows the significant (*p* value ≤ 0.05) associations of rs13330176*A for all available traits in the T1DKP, generated by bottom-line integrative analysis across all datasets in the Portal. The triangle data points indicate direction of effect and group-related traits on (*x*-axis) and −log10(*p*-value) of association is presented on the *y*-axis.

The last novel T1D association overlapping with an MS signal is localized in the *IRF8* gene region, the association led by the MS-protective variant, rs35703946*A, colocalized with a suggestive association with T1D, led by rs13330176*A (*p* = 5.5 × 10^−7^, OR = 0.93, 95% CI: 0.90–0.96, Figure 6) [2]. The latter variant replicates in an independent large study based on European samples [16] and was also associated with several quantitative traits and diseases (Figure 7).

These two lead variants are independent (r^2^ = 0.043) and have different functions. Notably, rs13330176*A has an opposite effect in MS (*p* = 1.195 × 10^−7^, OR = 1.12, 95%CI: 1.07–1.17) in the International Multiple Sclerosis Genetics Consortium dataset [17] compared with its protective effect in T1D [16]. The variant is located in a regulatory region within the H3K27Ac mark in the interferon regulatory factor 8 (*IRF8*) gene region.

#### 3.1.2. Novel MS Associations Colocalizing with Known T1D Signals

**Figure 8:**
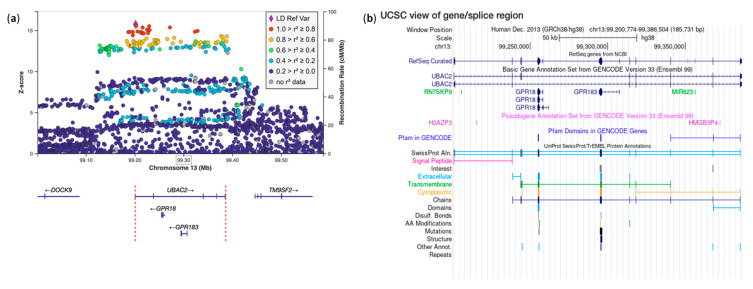
eQTL in the *UBAC2* gene and the genomic position of the *GPR18* and *GPR183* genes. (**a**) Regional association plot of the eQTL signal in the region of the *UBAC2* gene; each point represents a genetic variant plotted according to its genomic position (*x*-axis) and association Z-score (*y*-axis), where positive values indicate effect estimates in the direction of the tested allele and larger absolute values indicate stronger statistical evidence. The lead variant is shown as a purple diamond, while surrounding variants are colored based on their linkage disequilibrium (r^2^) with the lead variant, calculated using European samples from the 1000 Genomes Project. Recombination rates (cM/Mb) are shown as a blue line on the secondary *y*-axis. Gene annotations and transcriptional orientation are displayed below the association signals. Variant positions are based on the GRCh38 genome build. (**b**) UCSC Genome Browser view of the *UBAC2* gene region, according to the INTERVAL browser.

The novel variant rs9557185*C in the *UBAC2* gene was suggestively associated with MS risk in two studies (best *p* = 5.97 × 10^−7^, OR = 1.14, 95% CI: 1.08–1.20) [17,22]. This signal colocalized with an independent association with the same ancestry for T1D (r^2^ = 0.227, Supplementary Figure S6), led by the rs7332672*T allele, known to be associated with T1D (*p* = 1.29 × 10^−5^, OR = 0.94, 95% CI: 0.91–0.97) [16]. Both SNPs are in LD with an eQTL in PBMCS cells for the UBA Domain Containing 2 (*UBAC2*) gene (Figure 8), in peripheral blood mononuclear cells, namely rs34924388 CAGTT/C (*p*-value = 1.12 × 10^−54^, beta = 0.1191).

Variant rs4788115*A, located in the Linker for activation of T-cells family member (*LAT*) gene, is associated with T1D and suggestively with MS (*p* = 6.3 × 10^−7^, effect = 0.90, 95% CI: 0.86–0.94, Supplementary Figure S7) [22]. rs4788115*A increases the expression of the *LAT* gene in blood (*p* = 1.44 × 10^−12^; beta = 0.352; Open Targets Platform: https://platform.opentargets.org/variant/16_28986790_T_A, aceessed on 20 April 2026). This variant is located in a splice polypyrimidine tract in intron 6 of the gene and is a splicing QTL for *LAT*.

#### 3.1.3. SH2B3:p.Trp262Arg Known Overlapping Association with T1D and MS

One of the most interesting, previously reported overlapping associations between MS and T1D is located in the *SH2B3* gene, which encodes the SH2B adaptor protein 3, a negative regulator of hematopoiesis. The missense variant *SH2B3*:p.Trp262Arg (NP_005466; rs3184504; Table 2) results in loss of SH2B3 function, thereby promoting enhanced hematopoiesis. This variant has been associated with a wide range of traits and disorders, particularly autoimmunity, and is recognized as a shared genetic risk factor for both T1D and MS.

**Figure 9:**
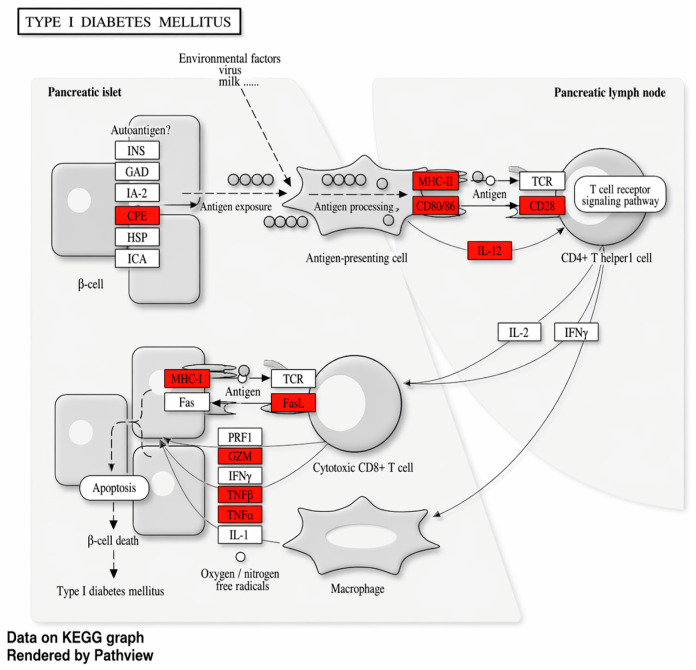
Representation of the T1D pathway based on KEGG. Proteins trans-regulated by the *SH2B3*:p.Trp262Arg variants are significantly enriched in the T1D pathway, as determined by the KEGG pathway analysis.

Using the UKB_PPP proteomic dataset (Open Targets Platform), we searched for the downstream effects of the *SH2B3*:p.Trp262Arg variant and identified 274 trans pQTLs. The set of trans-regulated proteins showed significant enrichment in multiple immune response-related pathways, including a notable enrichment in the T1D pathway (Figure 9). These findings suggest that the variant exerts widespread downstream effects on immune signaling networks, supporting its role in shared autoimmune mechanisms between T1D and MS.

### 3.2. Pathway Analysis of Colocalizing T1D-MS Associations

**Figure 10:**
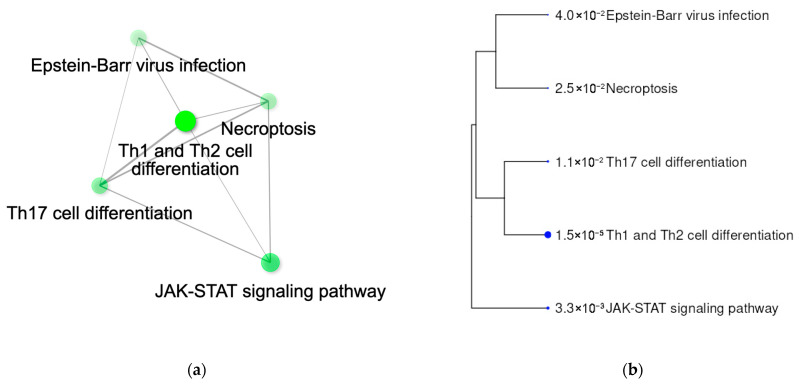
Relationship between enriched pathways resulting from the analysis of all colocalizing signals: (**a**) Plot showing the relationship between enriched pathways. Two pathways are considered connected if they share more than 20% of genes. Darker nodes are more significantly enriched gene sets. Bigger nodes represent larger gene sets. Thicker edges represent more overlapped genes. (**b**) A hierarchical clustering tree summarizes the correlation among significant pathways. Pathways with many shared genes are clustered together. Bigger dots indicate more significant *p*-values.

Global pathway analysis for all colocalizing genes confirmed that both disorders share the interferon-γ response (JAK-STAT pathway) to the pathogens, with a significant association with response to Epstein-Barr virus (Table 3). Furthermore, the enrichment pathway analysis showed that Th1, Th2, and Th17 lymphocytes are the immune cells primarily shared in T1D and MS predisposition. Interestingly, pathway analysis also yielded enrichment of genes in the necroptosis pathway (Table 2, Figure 10), which belongs to the programmed cell death pathways, such as apoptosis, pyroptosis, and necroptosis. They induce inflammation in response to pathogens or sterile stimuli, underscoring the T1D and MS etiopathology inflammatory component and thus therapeutic attention to constrain inflammation. In line with this, inhibition of cell death as a potential therapeutic strategy in inflammatory disorders was proposed [34].

## 4. Discussion

We colocalized large datasets of T1D and MS genetic signals and used other publicly available data to confirm associations and interpret the significance of novel signals. We postulate that T1D and MS share general autoimmunity loci rather than disease-specific loci, and often these shared signals act in opposite directions of effects in the two disorders, consistent with their independent etiopathology.

Risk variants for T1D are principally non-coding signals that, when cross-matched with epigenomic data, are enriched within lymphoid enhancers [29]. Accordingly, our study finds new key immune transcription factors associated with T1D—EOMES and IRF8. One study showed that risk variants for T1D were enriched in cis-regulatory elements, which were active in T cells as well as in non-immune cell types, including acinar and ductal cells of the exocrine pancreas [16]. Furthermore, risk variants at multiple T1D loci overlapped with exocrine-specific gene expression [16], supporting their contribution to T1D pathogenesis.

Searching for new targets and biomarkers, we performed a recently introduced method of in silico functional annotation of newly and suggestively associated variants.

One particularly interesting locus identified in our analysis was *EOMES*—encoding a master transcription factor that controls key checkpoints of maturation of natural killer cells, important in antiviral response [35,36,37]. Natural killer cells are key players in the innate immune response, and increasing evidence supports their involvement in the pathogenesis of T1D. Indeed, their frequency and function are altered in T1D patients, often showing reduced numbers and decreased cytotoxicity, particularly in long-standing disease [38,39]. *EOMES* is involved in the development of the central nervous system. It is also considered a master regulator of cytotoxic and natural killer cells and is essential for their expansion and maintenance, thereby having a pivotal role in both adaptive and innate immune responses. Eomes deficiency in CD8+ T cells resulted in the impaired expansion of these cells associated with decreased immune control of leukemia in mice [40]. This suggests that decreased expansion of CD8+ T cells has opposite effects in T1D and MS [41].

Another novel variant associated with T1D is located in the *IRF8* gene. *IRF8* is expressed at very high levels in mononuclear phagocytes and regulates the differentiation of granulocytes and macrophages, and the development of dendritic cells. Patients with immunodeficiency caused by pathogenic mutations in *IRF8* present a lack of circulating monocytes, dendritic cells, and basophils, but also present severe neutrophilia and eosinophilia [42]. In children with *IRF8* deficiency, severe depletion of antigen-presenting cells has been described in children with *IRF8* deficiency [42]. Mice mutant for Irf8 are susceptible to infection with intramacrophagic pathogens and hypersusceptible to *Mycobacterium*, viral, and fungal infections [42]. Selective pressure on variants that protect against *Mycobacterium tuberculosis* has been described [20]. Furthermore, *IRF8*-/- iPS cells showed reduced MHC class II expression and were impaired in cytokine responses, migration, and antigen presentation [43]. IRF proteins play central roles in controlling the expression of the IFN-α and IFN-β regulatory genes induced by viral infection. In particular, IRF8 binds the upstream regulatory region of type I interferon (IFN) and IFN-inducible MHC class I genes, playing a negative regulatory role in immune cells. These observations suggest that repressing the IFN pathway may have an opposite effect on T1D and MS during viral infection. In agreement with the function of *IRF8*, rs13330176 is a multiple trans eQTL (Open Targets Platform), downregulating downstream genes in the interferon pathway, many of which are located in the *HLA* region. It also regulates other genes, such as *FGD2*, expressed in B lymphocytes, macrophages, and dendritic cells (Open Targets Platform). Furthermore, it was proposed to play a role in leukocyte signaling and vesicle trafficking in antigen-presenting cells [44]. rs13330176 is also a trans eQTL for HLA class II genes (*DPA1*, *DQA1*, *DRA*, *DRB1*, *DPB1*, *DOB*), which play a central role in the immune system, presenting peptides derived from extracellular proteins. The variant also regulates other genes in trans (*COL8A2*, *NAPSB*, *PON2*) (Open Targets Platform), which contribute to the protection against oxidative stress.

Thus, it could be inferred that reduced antigen presentation may increase susceptibility to infection, and, unexpectedly, this mechanism exerts opposite effects in T1D and MS. This observation further supports our hypothesis that the genetic variation influencing T1D and MS often has opposite effects on shared pathways.

In the *UBAC2* gene region, the MS-associated variant mainly changes the expression levels of the G-Protein Coupled Receptor 18 (*GPR18*) gene in different immune subpopulations according to the Open Targets Platform browser (T-helper 1-2-17, CD8+ T, naïve regulatory T, T follicular helper, central memory CD4+ cells), supporting a role of the *GPR18* gene in T-cell regulation. In contrast, the T1D-associated variant mainly changes the expression levels of the *UBAC2* gene in whole blood and in CD14+ CD16- (classical monocytes) after 24-h stimulation with IFN-γ according to the Open Targets Platform browser. *GPR18* is expressed in microglia and binds Resolvin D2 (RvD2), which acts in the resolution of acute inflammation, regulating sepsis and bacterial clearance [45]. Conversely, *UBAC2* contributes to lymphopoiesis and lymphoid cell activation in the Wnt/beta-catenin pathway [46].

Considering the novel associations with MS, we detected a signal in the LAT gene encoding a molecule required for T-cell receptor (TCR) and pre-TCR-mediated signaling, in mature T cells and during their development. LAT is expressed in the thymus, T-cells, NK cells, mast cells, and at lower levels, in the spleen. Mutations in the *LAT* gene cause inborn errors of immunity in cases of SCID by pre-TCR/TCR signaling [47]. LAT contributes to FCGR3-mediated signaling in natural killer cells and FCER1-mediated signaling in mast cells, both implicated in MS pathogenesis [48]. The molecule encoded by this gene is required for T-cell receptor (TCR) and pre-TCR-mediated signaling, both in mature T cells and during their development. Mutations in the *LAT* gene cause inborn errors of immunity cases of Severe Combined Immunodeficiency (SCID) by pre-TCR/TCR signaling [47].

Our analysis also confirmed the central role of the general autoimmunity gene/coding variant in *SH2B3*-rs3184504 associated with both disorders. The effect of this variant is substantial in T1D, while it is smaller in MS.

Our global pathway analysis for all colocalizing genes underlined the importance of the interferon-γ response (JAK-STAT pathway) to pathogens, and a significant association with response to Epstein-Barr virus in both disorders. Epstein-Barr virus has been extensively studied in MS, but this enrichment is rather novel for T1D. The enrichment of genes in the necroptosis pathway, which belongs to the programmed cell death pathway, is in line with the interferon pathway involvement in both disorders. This highlights that the T1D and MS inflammatory component is triggered by pathogens. Thus, therapeutic attention must be taken to detect reactivations or infections in order to constrain inflammation. Furthermore, the inhibition of cell death as a potential therapeutic strategy in inflammatory disorders was proposed.

Previous reports underline several similarities shared between T1D and MS in clinical, epidemiological, and immunological features, suggesting a common mechanism of disease development [49]. However, there have been no detailed reports describing such similarities. Our study showed that the shared genetics of different autoimmune pathologies mirrors the shared etiology of the diseases. In addition, we observed differences between T1D and MS that uncover disease pathways and mechanisms, providing insights into each disease’s pathogenesis. This helps explain differential collateral effects on monoclonal antibody therapy and drug repurposing.

Overlapping genetic associations have been reported across multiple autoimmune disorders, with previous GWASs on autoimmunity identifying shared association regions, including T1D and MS. In our study, we also found shared signals between these two pathologies; however, genetic colocalization showed that some of these signals represent independent associations in the same gene, indicating the presence of genetic heterogeneity. Additionally, some of the shared signals have opposite effects in the two diseases, highlighting divergent pathological mechanisms.

By intersecting the shared variants between T1D and MS with data from other autoimmune disorders, we inferred that T1D and MS are characterized by common immune-related loci but do not share private, disease-specific signals or characteristics of each disease. This is consistent with previous reports and indicates that, while shared pathogenic mechanisms are common, there is no single global mechanism that has been characteristic of autoimmune polygenic disorders [1].

Although several signals show opposite directions of effects, pointing to the differences between T1D and MS, several “general autoimmunity” genes/variants with the same direction of effects were described, including *TYK2*, *SH2B3*, *PTPN22*, and *BACH* [1,50]. Three of which were also detected by our analysis.

The increasing evidence in literature supports that both T1D and MS are heterogeneous disorders consisting of multiple clinical subtypes with distinct pathophysiological features. The age of onset differs for both disorders, and gender differences have been observed, with males having a higher prevalence in T1D and females having a higher prevalence in MS [51].

As an explanation of opposite effects, genetic sharing of the etiopathology in autoimmune disorders has been characterized, emphasizing the action of dysregulation of key autoimmune genes, and mirroring the evolutionary forces of human immune system adaptation to different pathogens during the waves of human migrations and admixtures [52].

This study has several limitations. First, the analyses were largely restricted to populations of European ancestry, which may limit the generalizability of the findings. Second, we relied on discovery-stage, non-final MS GWAS summary statistics and considered suggestive significance thresholds rather than genome-wide significance. Third, the study lacks formal replication in an independent cohort with pre-specified criteria. Fourth, colocalization analyses alone cannot establish causality. Fifth, the use of default prior probabilities in the colocalization framework may influence posterior probabilities and, consequently, the inference of shared genetic signals. Finally, this study is bioinformatic and has no new clinical data to confirm the results. Therefore, our findings should be interpreted with these limitations in mind.

Understanding the similarities and differences in etiopathology and genetic architecture between these two autoimmune disorders will contribute to the understanding of the causal mechanisms and treatments for T1D and MS.

## 5. Conclusions

In summary, our study highlights common genetic signals in T1D and MS and infers immune heterogeneity between these two autoimmune disorders, which are often characterized by antagonistic pleiotropy. This phenomenon may be important in drug target repurposing and helpful in explaining the autoimmune adverse events of monoclonal antibodies in T1D and MS.

We show that even if part of the loci is shared, the direction of effects is opposite-this points to the immunological heterogeneity of the autoimmune disorders. Thus, the differences in the shared pathways.

## Figures and Tables

**Table 1:** Similarities and differences between T1D and MS.

	Multiple Sclerosis	Type 1 Diabetes
**Immune background**	Immune-mediated	Autoimmune
**Clinical Age of onset**	Usually young adults (peak 20–40 years), but pediatric and late-onset forms exist	Often childhood
**Organ-specificity**	CNS (brain, spinal cord, optic nerve)	Pancreatic β-cells
**Type of therapy**	Disease-modifying immunotherapy	Insulin replacement; emerging disease-modifying immunotherapy
**Immune cell contribution**	Cell mediated	Cell mediated
**Genetic background—HLA**	DR15	DR3, DR4
**Autoantibodies**	No characteristic	Serum insulin autoantibodies IAA, GADA, IA2A, ZnT8A
**Pathogens**	EBV	Enteroviruses (?)
**Triggering factors**	EBV, low vitamin D/sunlight, smoking, adolescent obesity, other environmental factors	Viral infections, microbiome and early-life environmental factors
**World distribution**	Longitudinal gradient	Longitudinal gradient
**Seasonality of the onset**	Reported	Reported
**Sex distribution**	Female predominance (~2–3:1)	Slightly male predominance
**Disease course**	Relapsing Remitting, Progressive	No subtyping

**Table 2:** The colocalizing signals between T1D and MS.

Gene/Region	Chromosome Position	MS Lead	T1D Lead	Direction of Effects	r^2^ Betweenlead SNPs	Locus H4	Novelty
*EOMES*	3p24.1	rs13327021*C	rs3806624*A	opposite	0.6845	0.8770	T1D novel
*RGS14 */PRLD1	5q35.3	rs67111717*A	rs4073745*A	opposite	0.4047	0.8499	T1D novel
*DLL1 */FAM120B	6q27	rs2273215*G	rs1075163*G	opposite	0.0249	0.8454	T1D novel
*ZNF438 */ZEB1	10p11.22	rs1087056*G ^1^	rs2066250*C ^1^	opposite	0.2378	0.9460	T1D novel
*SESN3 */MTMR2	11q21	rs4409785*C	rs11021232*C	same	0.9527	0.8859	T1D Novel
*WARS1 */SLC25A47	14q32.2	rs8009765*A	rs8015259*G	same	0.9558	0.7422	T1D novel
*IRF8*	16q24.1	rs35703946*A ^1^	rs13330176*A ^1^	same	0.0432	0.7632	T1D novel
*UBAC2*	13q32.3	rs9557185*C	rs7332672*T	opposite	0.2267	0.7721	MS Novel
*LAT*	16p11.2	rs4788115*A	rs4788115*A	opposite	1	1	MS novel
*RUNX3*	1p36.11	rs6672420*A	rs10751776*A	opposite	0.8850	0.9957	known
*RGS2-AS1*	1q31.2	rs1323292*A	rs10801128*G	same	0.5241	0.9369	known
*ANKRD55*	5q11.2	rs7731626*A	rs71624119*A	same	0.5239	0.9837	known
*BACH2*	6q15	rs72928038*A	rs6908626*T	same	0.9525	0.9925	known
*IL20RA*/*IL22RA2*/*IFNGR1*	6q23.3	rs631204*A	rs12665429*C	opposite	0.2027	0.8631	known
*WAKMAR2*/TNFAIP3	6q23.3	rs17779870*T	rs4548024*T	same	0.6004	0.9483	known
*TAGAP*	6q25.3	rs1738074*C	rs182429*G	same	0.9924	0.9930	known
*SKAP2*/HOXA	7p15.2	rs11563327*T	rs2253415*G	same	0.9273	0.8098	Known T1D
*IKZF1*	7p12.2	rs7385730*T	rs876039*C	same	0.4647	0.9683	known
*ZMIZ1*	10q22.3	rs1250551*G	rs1782648*G	same	0.8885	0.9646	known
*PRDX5*/CCDC88B	11q13.1	rs11231749*T ^1^	rs663743*A ^1^	same	0.2248	0.9531	known
*CLECL1P*	12p13.31	rs7977720*C	rs10844597*G	opposite	0.8484	0.9653	known
SH2B3	12q24.12	rs3184504*T	rs3184504*T	same	1	0.9684	known
CLEC16A	16p13.13	rs1364254*A	rs8063318*G	same	0.8483	0.9578	known
RMI2	16p13.13	rs28693671*T	rs13334203*A	same	1	1	known
IKZF3/ZPBP2/GSDMB	17q12	rs9909593*A	rs12950209*T	same	0.8147	0.8697	known
PDE4A/TYK2	19p13.2	rs2043330*T	rs34536443*C	same	0.065	0.9147	known

^1^ Locus heterogeneity.

**Table 3:** Pathway analysis of all colocalizing signals between T1D and MS (FDR < 0.05).

Pathway	Enrichment FDR	Fold Enrichment	Genes	Pathway Genes
Th1 and Th2 cell differentiation	1.49 × 10^−5^	36.11	*LAT, DLL1, IFNGR1, TYK2, RUNX3*	92
JAK-STAT signaling pathway	3.33 × 10^−3^	15.82	*IL22RA2, IFNGR1, IL20RA, TYK2*	168
Th17 cell differentiation	1.08 × 10^−2^	18.63	*LAT, IFNGR1, TYK2*	107
Necroptosis	2.53 × 10^−2^	12.53	*IFNGR1, TNFAIP3, TYK2*	159
Epstein-Barr virus infection	3.99 × 10^−2^	9.87	*TNFAIP3, TYK2, RUNX3*	202

## Data Availability

All data are included within the text of the manuscript.

## Supplementary Materials

The following supporting information can be downloaded at: https://www.mdpi.com/article/10.3390/genes17050531/s1, Figure S1: Regional association plot for the RGS14/PRLD1 locus in MS (a) and T1D (b), Figure S2: Regional association plot for the DLL1/FAM120B locus in MS (a) and T1D (b), Figure S3: Regional association plot for the ZNF438/ZEB1 locus in MS (a) and T1D (b), Figure S4: Regional association plot for the SESN3/MTMR2 locus in MS (a) and T1D (b), Figure S5: Regional association plot for the WARS1/SLC25A47 locus in MS (a) and T1D (b), Figure S6: Regional association plot for the UBAC2 locus in MS (a) and T1D (b), Figure S7: Regional association plot for the LAT locus in MS (a) and T1D (b).

## Abbreviations

The following abbreviations are used in this manuscript:

T1D	Type 1 diabetes
MS	Multiple sclerosis
GWAS	Genome-Wide Association Studies
SNPs	Single Nucleotide Polymorphisms
HLA	Human Leucocyte Antigen
TYK2	Tyrosine Kinase 2
CLEC16A	C-Lectin 16A
PRF1	Perforin 1
eQTL	Expression Quantitative Trait Loci
pQTL	Protein Quantitative Traits Loci
NHGRI-EBI	National Human Genome Research Institute- European Bioinformatics Institute
IMSGC	International Multiple Sclerosis Genetics Consortium
RUNX3	RUNX family transcription factor 3
RGS2-AS1	RGS2 antisense RNA1
EOMES	Eomesodermin
ANKRD55	Ankyrin repeat domain 55
RGS14	Regulator of G protein signaling 14
PRLD1	PRELI Domain containing 1
BACH2	BACH transcriptional regulator 2
IL20RA	Interleukin 20 receptor subunit alpha
IL22RA2	Interleukin 22 receptor subunit alpha 2
IFNGR1	Interferon gamma receptor 1
WAKMAR2	Wound and keratinocyte migration-associated lncRNA 2
TNFAIP3	Tumor Necrosis Factor, Alpha-Induced Protein 3
TAGAP	T cell activation RhoGTPase-activating protein
DLL1	Delta-like canonical Notch ligand 1
FAM120B	Family with sequence similarity 120 member B
SKAP2	Src kinase-associated phosphoprotein 2
HOXA	Homeobox A1
IKZF1	ICAROS family zinc finger 1
ZNF438	Zinc finger protein 438
ZEB1	Zinc finger E-box binding homeobox 1
ZMIZ1	Zinc finger MIZ-type containing 1
PRDX5	Peroxiredoxin 5
CCDC88B	Coiled-coil and HOOK domain protein 88B
SESN3	Sestrin 3
MTMR2	Myotubularin-related protein 2
CLECL1P	C-type lectin-like 1
SH2B3	SH2B adaptor protein 3
UBAC2	UBA domain containing 2
WARS1	Tryptophanyl-tRNA synthetase 1
SLC25A47	Solute carrier family 25 member 47
RMI2	RecQ-mediated genome instability 2
LAT	Linker for activation of T cells
IRF8	Interferon regulatory factor 8
IKZF3	IKAROS family zinc finger 3
ZPBP2	Zona pellucida binding protein 2
GSDMB	Gasdermin B
PDE4A	Phosphodiesterase 4A
LD	Linkage Disequilibrium
OR	Odds Ratio
3’UTR	3’ Untranslated region
MAF	Minor Allele Frequency
CIS	Clinically Isolated Syndrome
TIGIT	T cell immunoreceptor with Ig and ITIM domains
GPR18	G protein—coupled receptor 18
IFN- γ	Interferon—gamma
RvD2	Resolvin D2
SCID	Severe Combined Immunodeficiency
TCR	T cell receptor
JAK-STAT	Janus Kinase—Signal Transducer and Activator of Transcription
Th1	Lymphocytes T helper 1
Th2	Lymphocytes T helper 2
Th17	Lymphocytes T helper 17
