# Supplementary material for: Type 1 Diabetes and Multiple Sclerosis Share General Autoimmunity Genetic Variation
Source: Genes (Basel). 2026 Apr 30;17(5):531. doi: 10.3390/genes17050531 (PMC13205159; doi:10.3390/genes17050531)
Supplement: Supplementary file 1 [file genes-17-00531-s001.zip › genes-4200872-supplementary.pdf]

**Supplementary Figures**

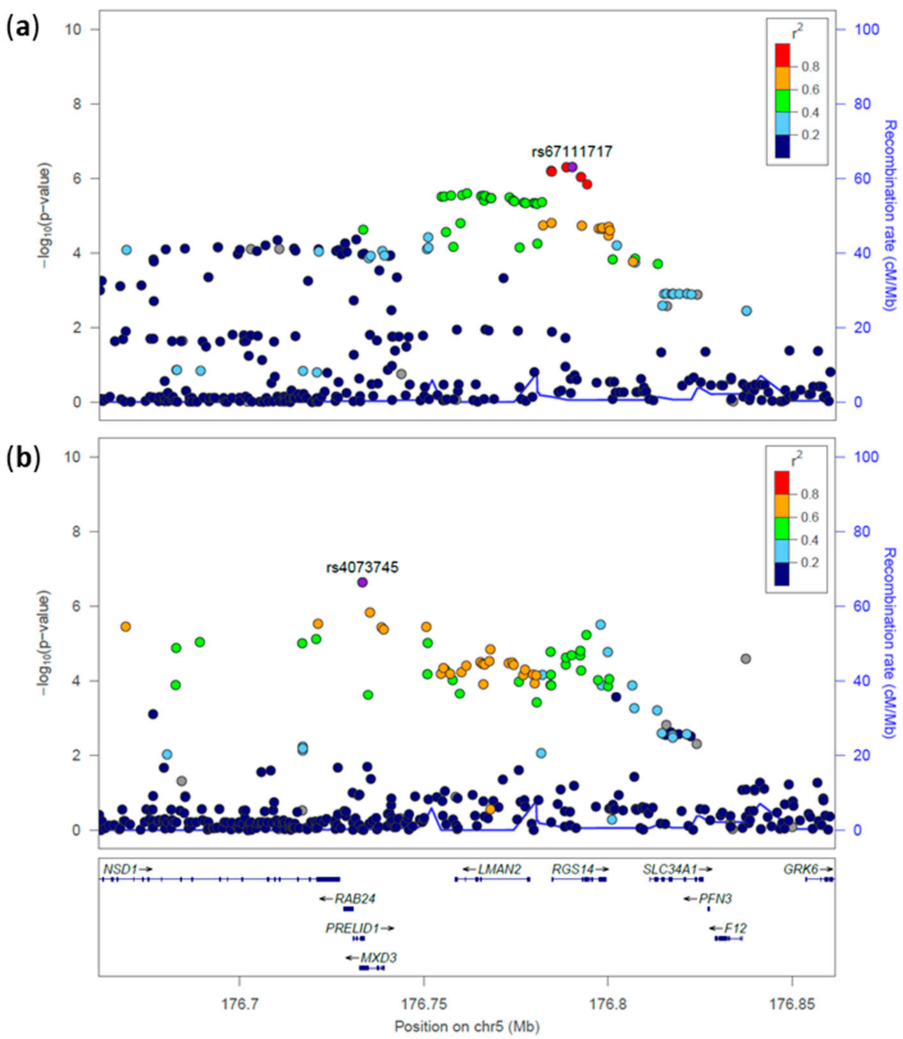


**Supplementary Figure S1. Regional association plot for the *RGS14/PRLD1* locus in MS (a) and T1D (b).** Each point represents a genetic variant plotted according to its genomic position (x-axis) and -log10(p-value) of association (y-axis). The lead variant is shown as a purple diamond, while surrounding variants are colored based on their linkage disequilibrium (LD;r2) with the lead variant, calculated using European samples from the 1000 Genomes Project. Recombination rates (cM/Mb) are shown as a blue line on the secondary y-axis. Gene annotations and transcriptional orientation are displayed below the association signals. Variant positions are based on the GRCh37 genome build.

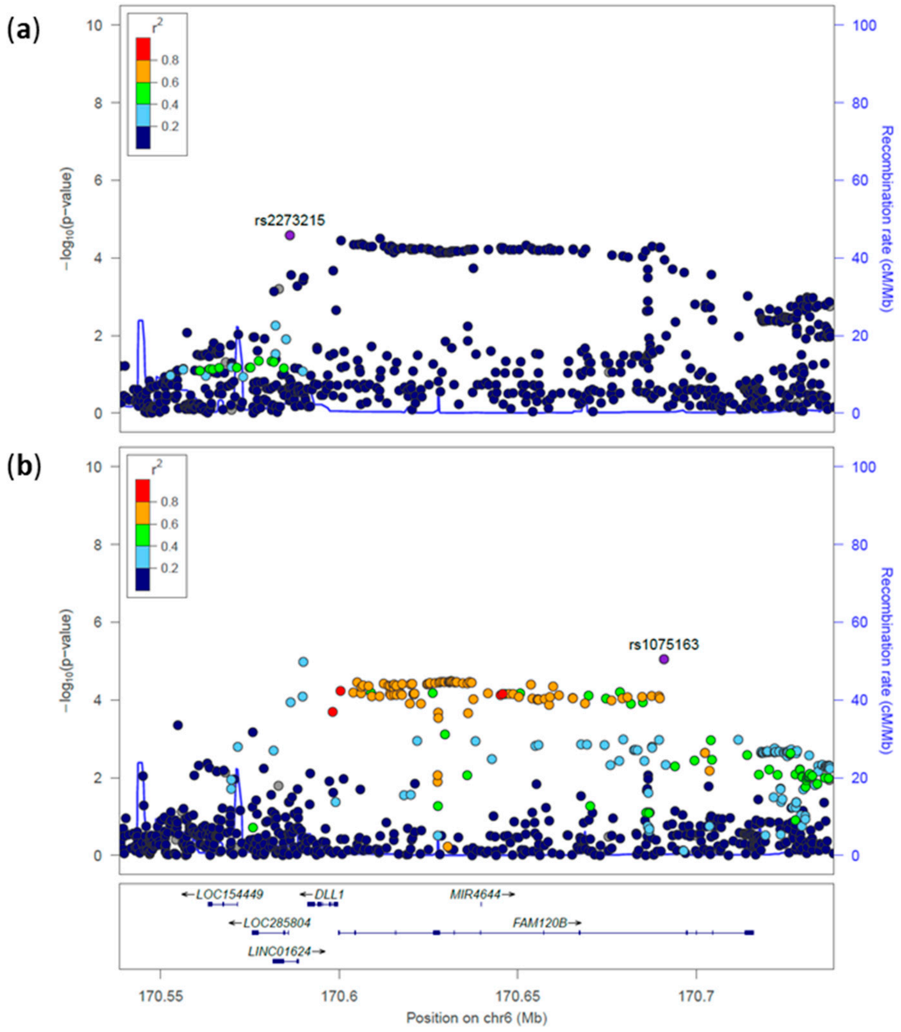


**Supplementary Figure S2. Regional association plot for the *DLL1/FAM120B* locus in MS (a) and T1D (b).** Plot details, including variant colored by linkage disequilibrium (LD), recombination rates, and gene annotations, are described in Supplementary Figure S1.

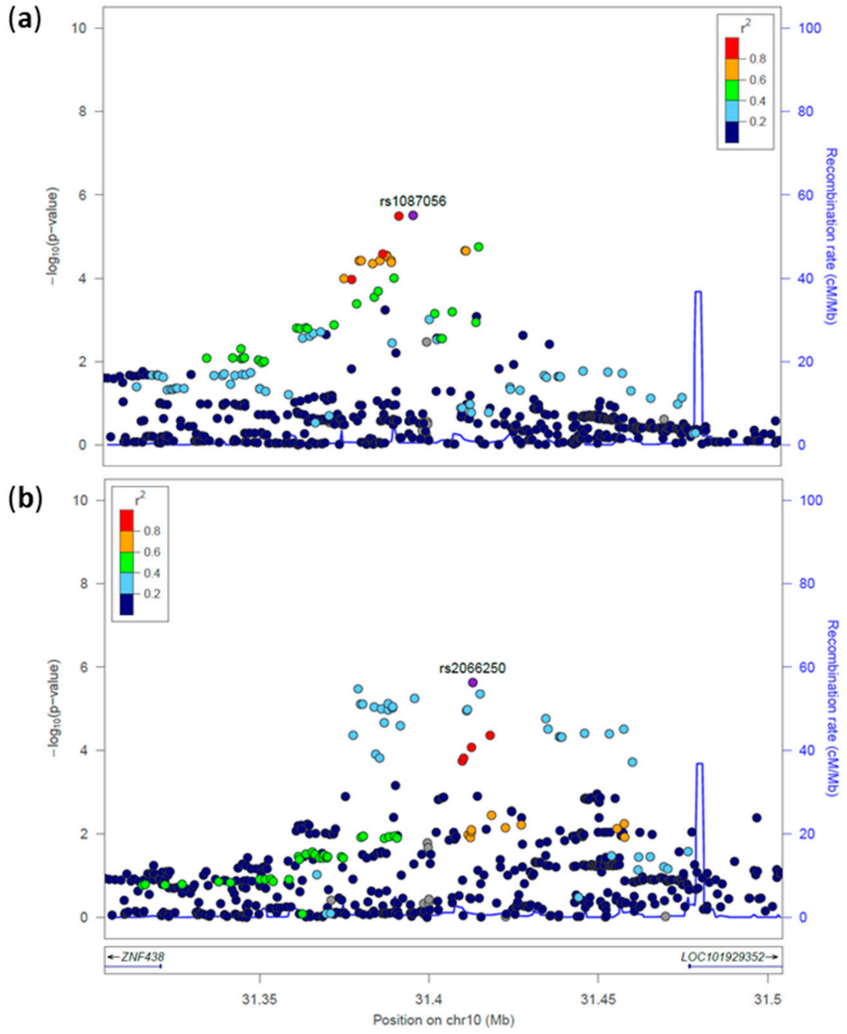


**Supplementary Figure S3. Regional association plot for the *ZNF438/ZEB1* locus in MS (a) and T1D (b).** Plot details, including variant colored by linkage disequilibrium (LD), recombination rates, and gene annotations, are described in Supplementary Figure S1.

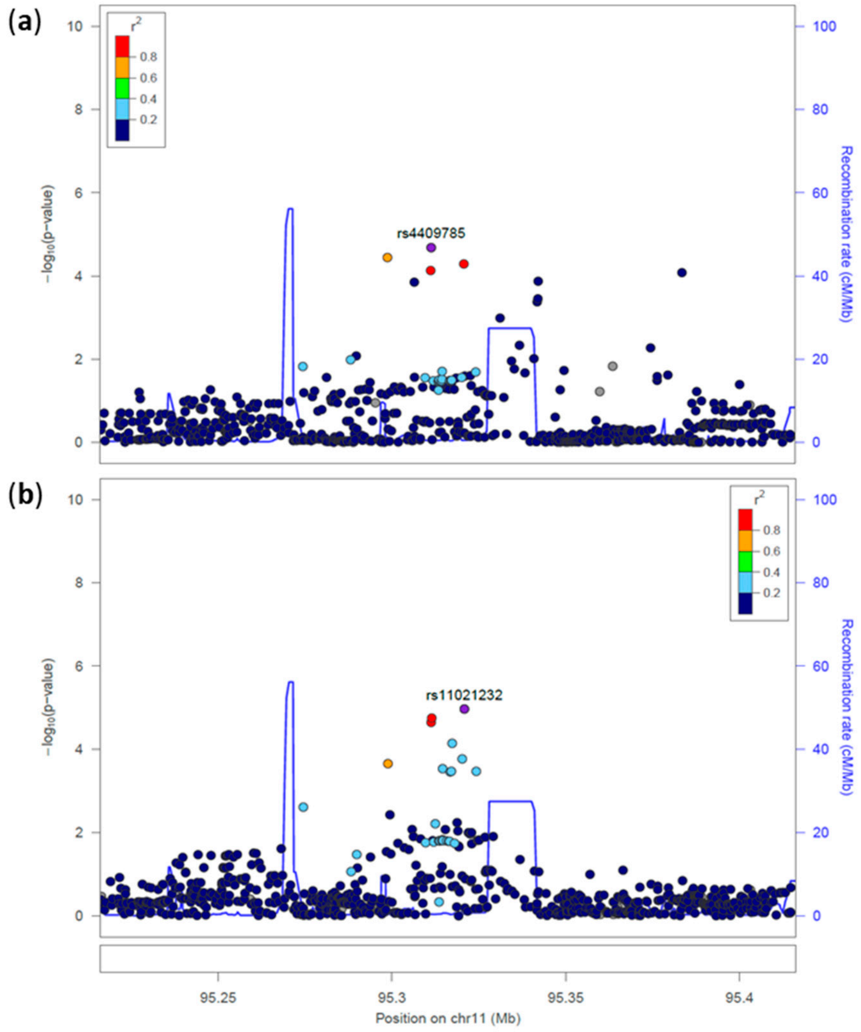


**Supplementary Figure S4. Regional association plot for the *SESN3/MTMR2* locus in MS (a) and T1D (b).** Plot details, including variant colored by linkage disequilibrium (LD), recombination rates, and gene annotations, are described in Supplementary Figure S1.

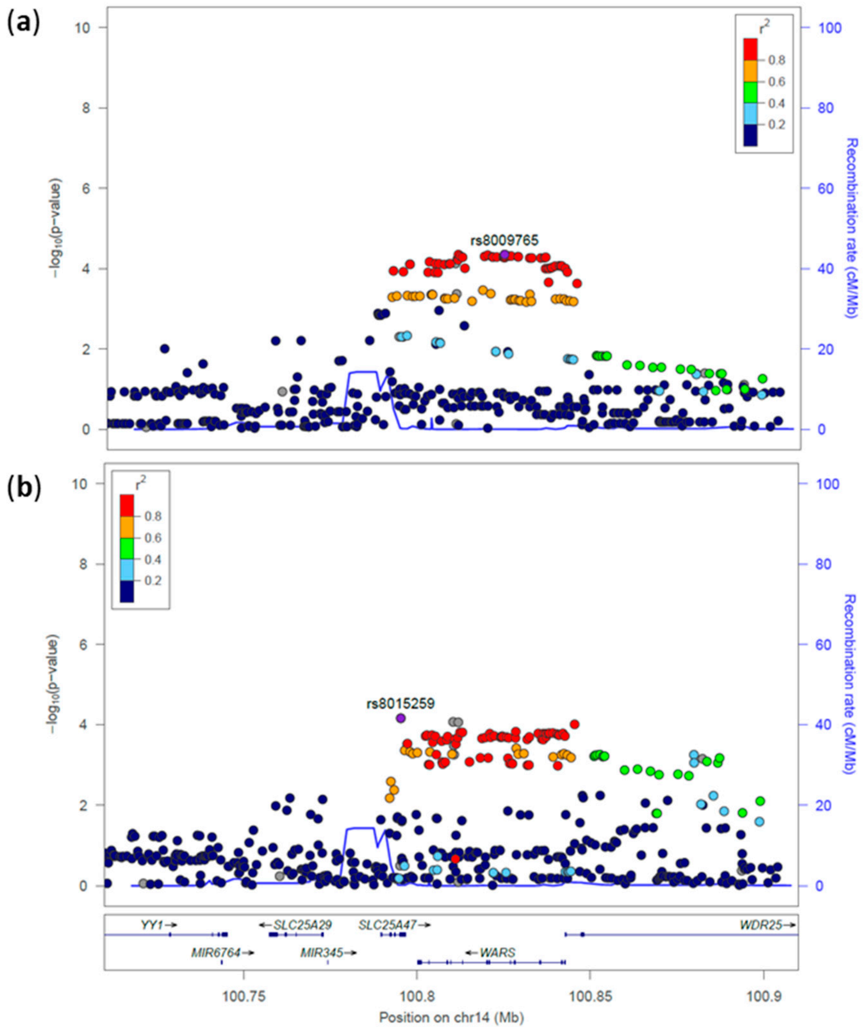


**Supplementary Figure S5. Regional association plot for the *WARS1/SLC25A47* locus in MS (a) and T1D (b).** Plot details, including variant colored by linkage disequilibrium (LD), recombination rates, and gene annotations, are described in Supplementary Figure S1.

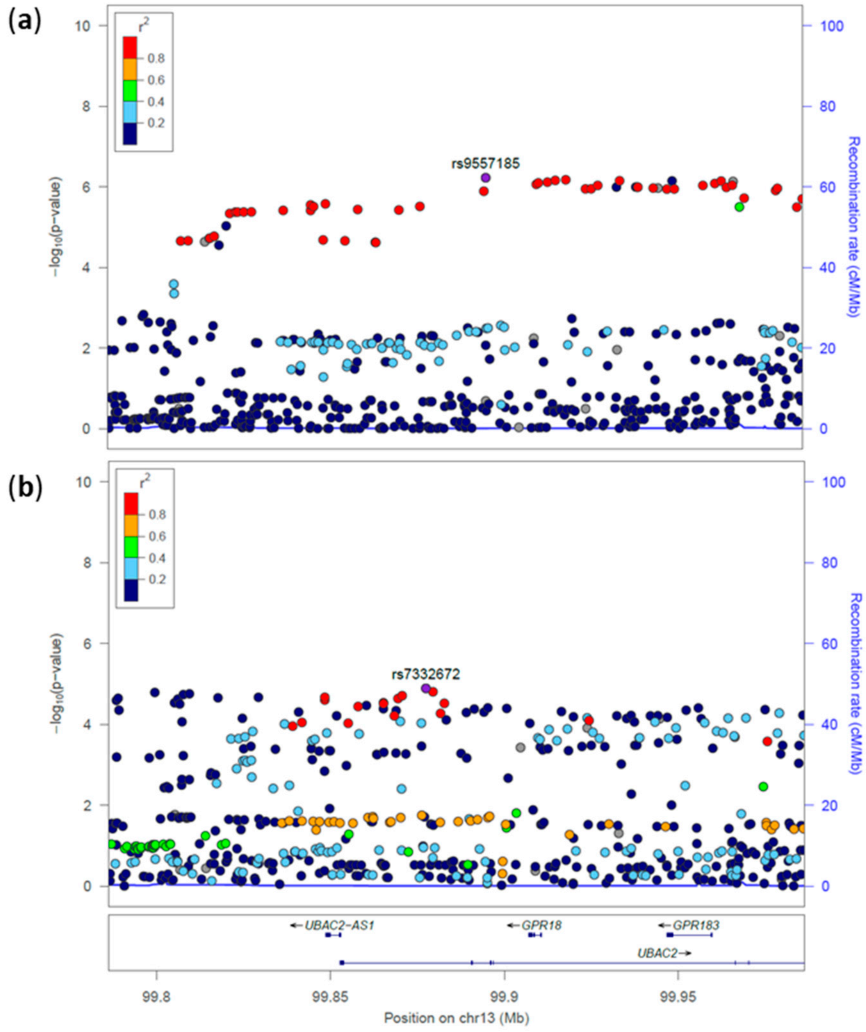


**Supplementary Figure S6. Regional association plot for the *UBAC2* locus in MS (a) and T1D (b).** Plot details, including variant colored by linkage disequilibrium (LD), recombination rates, and gene annotations, are described in Supplementary Figure S1.

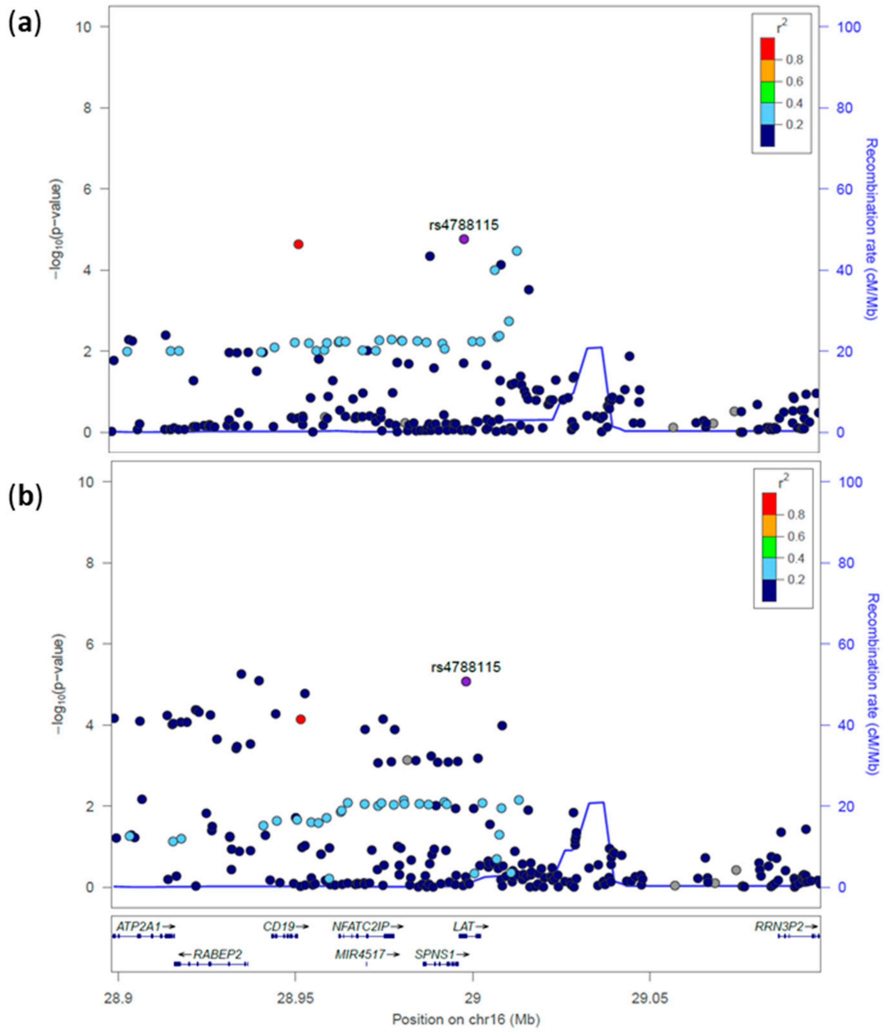


**Supplementary Figure S7. Regional association plot for the *LAT* locus in MS (a) and T1D (b).** Plot details, including variant colored by linkage disequilibrium (LD), recombination rates, and gene annotations, are described in Supplementary Figure S1.
